# Harmonisation of short-term in vitro culture for the expansion of antigen-specific CD8^+^ T cells with detection by ELISPOT and HLA-multimer staining

**DOI:** 10.1007/s00262-014-1593-0

**Published:** 2014-08-19

**Authors:** Lindsey Chudley, Katy J. McCann, Adam Coleman, Angelica M. Cazaly, Nicole Bidmon, Cedrik M. Britten, Sjoerd H. van der Burg, Cecile Gouttefangeas, Camilla Jandus, Karoline Laske, Dominik Maurer, Pedro Romero, Helene Schröder, Linda F. M. Stynenbosch, Steffen Walter, Marij J. P. Welters, Christian H. Ottensmeier

**Affiliations:** 1 Cancer Sciences Unit, Faculty of Medicine, Experimental Cancer Medicine Centre, Southampton General Hospital, University of Southampton, Tremona Road, Southampton, SO16 6YD UK; 2 Translational Oncology, University Medical Center, Johannes-Gutenberg University GmbH, Mainz, Germany; 3 Department of Clinical Oncology, Leiden University Medical Centre, Leiden, The Netherlands; 4 Department of Immunology, Institute for Cell Biology, Eberhard-Karls University, Tübingen, Germany; 5 Translational Tumour Immunology, Ludwig Institute for Cancer Research, Lausanne, Switzerland; 6 Immatics Biotechnologies GmbH, Tübingen, Germany; 7 Somers Cancer Research Building (Mailpoint 824), Cancer Sciences Unit, Faculty of Medicine, Southampton General Hospital, University of Southampton, Tremona Road, Southampton, SO16 6YD UK

**Keywords:** T cell, In vitro stimulation, ELISPOT, Multimer, Harmonisation, Inter-laboratory testing

## Abstract

Ex vivo ELISPOT and multimer staining are well-established tests for the assessment of antigen-specific T cells. Many laboratories are now using a period of in vitro stimulation (IVS) to enhance detection. Here, we report the findings of a multi-centre panel organised by the Association for Cancer Immunotherapy Immunoguiding Program to investigate the impact of IVS protocols on the detection of antigen-specific T cells of varying ex vivo frequency. Five centres performed ELISPOT and multimer staining on centrally prepared PBMCs from 3 donors, both ex vivo and following IVS. A harmonised IVS protocol was designed based on the best-performing protocol(s), which was then evaluated in a second phase on 2 donors by 6 centres. All centres were able to reliably detect antigen-specific T cells of high/intermediate frequency both ex vivo (Phase I) and post-IVS (Phase I and II). The highest frequencies of antigen-specific T cells ex vivo were mirrored in the frequencies following IVS and in the detection rates. However, antigen-specific T cells of a low/undetectable frequency ex vivo were not reproducibly detected post-IVS. Harmonisation of the IVS protocol reduced the inter-laboratory variation observed for ELISPOT and multimer analyses by approximately 20 %. We further demonstrate that results from ELISPOT and multimer staining correlated after (*P* < 0.0001 and *R* ^2^ = 0.5113), but not before IVS. In summary, IVS was shown to be a reproducible method that benefitted from method harmonisation.

**Electronic supplementary material:**

The online version of this article (doi:10.1007/s00262-014-1593-0) contains supplementary material, which is available to authorized users.

## Introduction

Accurate assessment of both frequency and functionality of T cells to allow reproducible immune monitoring has been the focus of many laboratories in a wide range of fields [1–4]. The methods used for T-cell enumeration, i.e. ELISPOT assay and multimer staining, have been the subject of a number of harmonisation studies, in particular in the fields of infectious diseases [1, 5] and cancer, where large proficiency panels have been conducted both in Europe and the United States within the remit of the Association for Cancer Immunotherapy Immunoguiding Programme (CIMT-CIP) [6] and the Cancer Immunotherapy Consortium of the Cancer Research Institute (CIC-CRI) [7], respectively. Such panels have investigated factors that influence the frequency of responses detected by ex vivo IFNγ ELISPOT [6, 8, 9] and multimer staining [10, 11].

More recently laboratories have incorporated a period of in vitro stimulation (IVS) prior to assay by ELISPOT or multimer staining. Several studies have shown that ex vivo IFNγ ELISPOT and cultured ELISPOT measure different subsets of effector and memory T cells [12, 13]. Whilst the ex vivo ELISPOT quantifies effector cells, the cultured ELISPOT can measure T cells of central memory phenotype, which are able to proliferate and consequently acquire effector function [14]. IVS for as little as 1 day, when followed by peptide stimulation for 24–48 h in an ELISPOT assay, has been shown to enhance cellular responses to *M. tuberculosis* [4]. We have also shown in a DNA vaccine clinical trial in patients with prostate cancer that vaccine peptide-specific responses detectable by ELISPOT could be increased by 33 % following IVS for 9 days prior to assay; 6/30 versus 16/30 responders for ex vivo and cultured ELISPOT, respectively [15]. Hence, IVS provides an effective method to enhance the detection of antigen-specific T-cell populations.

Since laboratories have developed their in-house IVS techniques independently, a diverse set of assay parameters are in use regarding cell concentration/density, length of culture, peptide concentration and type and number of exogenous cytokines, among others [16]. An understanding of how comparable and robust individual IVS methods, including a correlation of results post-culture with ex vivo frequencies, is required to better enable the interpretation of data generated following IVS of PBMCs.

Here, we describe our findings from a 2 stage harmonisation process that examined the robustness and variability of short-term in vitro culture for the expansion of antigen-specific T cells of varying ex vivo frequencies. We first evaluated the ability of 5 distinct IVS protocols, originating from 5 laboratories across Europe, to detect pre-defined antigen-specific responses in multiple donors by ELISPOT assay and multimer staining. Features of the “best-performing” IVS method(s) were integrated to establish a harmonised protocol that was then used in each centre to further evaluate inter-assay (for each centre) and inter-laboratory variation in a second phase of the study.

## Materials and methods

The following Materials and Methods section is MIATA compliant (www.miataproject.org) [17]; further details of each centre’s reagents and protocols are provided in Supplementary MIATA Information.

### Organisation and panel design

**Fig. 1:**
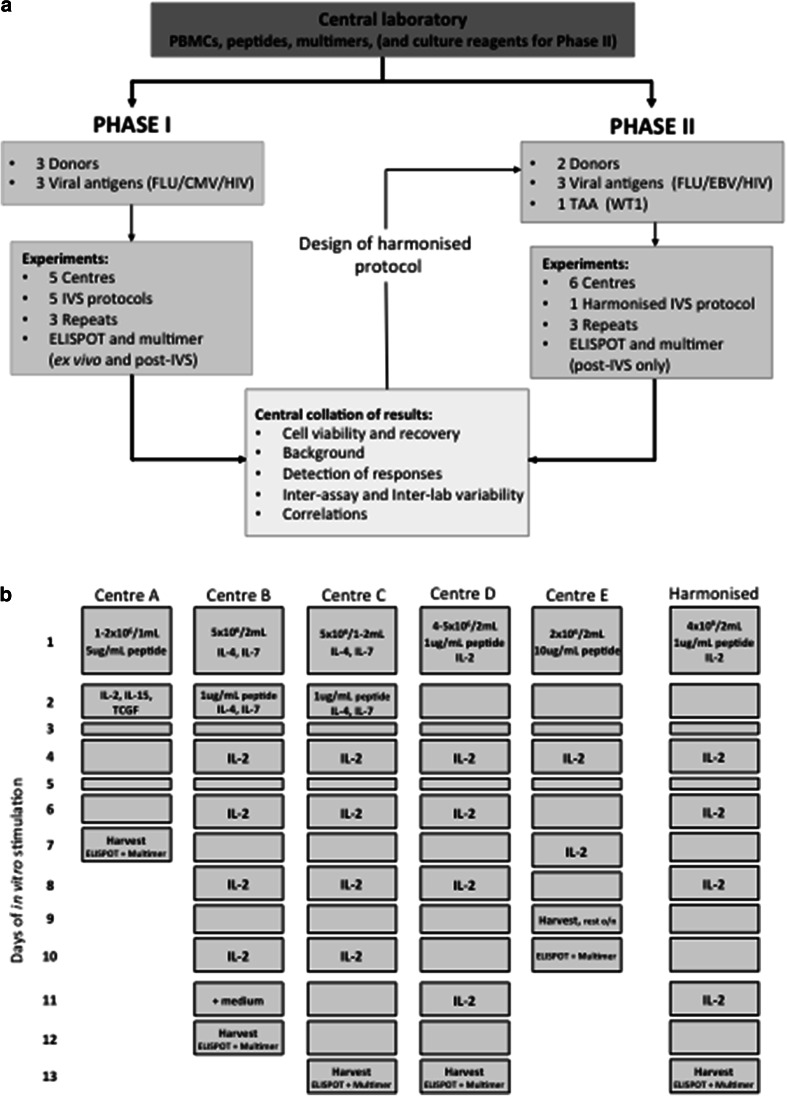
A multi-centre, 2 phase in vitro stimulation proficiency panel An overview of the design of the IVS proficiency panel (**a**). A schematic representation of the 5 IVS protocols used by participating centres (*A*–*E*) in Phase I and the harmonised IVS protocol used by 6 centres (*A*–*F*) in Phase II (**b**)

The proficiency panel was conducted in 2 phases with 5 and 6 centres participating in Phase I and II, respectively, from 4 European countries (UK, Germany, The Netherlands and Switzerland). The panel design is shown in Fig. 1a.

*Phase I:* Five centres received centrally prepared PBMCs from 3 HLA-A2^+^ donors, peptides (Peptide Synthetics Peptide Protein Research Ltd., Bishops Waltham, UK.) and multimers (kindly supplied by the Department of Immunology, Institute for Cell Biology, Eberhard-Karls University Tübingen) sufficient to perform the requested assays. Centres were required to perform (i) ex vivo ELISPOT and multimer analysis and (ii) IVS, according to the centre’s own established protocol (Fig. 1b and Supplementary MIATA Information, IVS Module 2), followed by ELISPOT and multimer analysis, for defined antigens. IVS was to be performed on 3 occasions, with culture set up on different days. Results were reported back to the organising centre for analysis. Features of the “best-performing” IVS protocol(s) were identified and used to establish a harmonised protocol for further testing in a second phase.

*Phase II:* Six centres received centrally prepared PBMCs from 2 HLA-A2^+^ donors, peptides (Peptide Synthetics Peptide Protein Research Ltd), multimers (production in-house), X-Vivo 15 medium (Lonza Group Ltd., Basel, Switzerland), l-glutamine (PAA Laboratories Ltd., Yeovil, UK.), Pen/Strep (PAA), human AB serum (Lonza Group Ltd.) and recombinant IL-2 (R&D Systems Europe Ltd., Abingdon, UK.). As for Phase I, centres were required to perform harmonised IVS (Fig. 1b) on 3 occasions, each followed by ELISPOT and multimer staining. Results were reported back to the organising centre for analysis.

### Donor PBMC and pre-screening

PBMCs were isolated from anonymised buffy cones (HIV status negative) obtained from the National Blood Service, Southampton University Hospitals NHS Foundation Trust, as previously described [6]. PBMC aliquots were stored in liquid nitrogen until shipment on dry ice to participating centres, where they were returned to liquid nitrogen until required.

Pre-screening of donor PBMCs to ensure consistent viability and recovery upon thawing and to identify donors with suitable T-cell reactivity to the HLA-A*0201-restricted epitope peptides was performed at the organising centre. T-cell reactivity to defined viral and tumour-associated antigens (TAA) (Supplementary Table 1) was determined by ex vivo and post-IVS IFNγ ELISPOT assay and multimer analysis (triplicate). T-cell reactivity was defined as high (≥ 100 spot forming cells (SFC)/million), intermediate (50–100 SFC/million) or low (21–50 SFC/million) using ex vivo ELISPOT data alone; furthermore, responses were characterised as low/undetectable if ex vivo ELISPOT was ≤ 20 SFC/million, but for which ex vivo multimer staining could detect a positive, but low, population of multimer^+^ T cells. The serology status of the donors to the specific viral antigens was unknown.

### Detection of antigen-specific T-cell responses

Ex vivo (Phase I only) and post-IVS antigen-specific T-cell responses were assessed by IFNγ ELISPOT assay and multimer staining; if cell number was limited, cells were prioritised for ELISPOT over multimer staining.

*IFNγ ELISPOT:* For Phase I, a PBMC resting period prior to ex vivo ELISPOT was mandated; otherwise, each centre performed the ELISPOT assay according to their own established protocol (Supplementary MIATA Information, ELISPOT Module 2). Recommendations for peptide concentration and cell number were 1 μg/mL (ex vivo and post-IVS) and 4 × 10^5^ cells per well (ex vivo, test and control wells), respectively. Centres supplied their own reagents throughout, with the exception of peptides. Post-IVS ELISPOT assay set up was more tightly regulated in Phase II, with mandatory requirements: (i) all steps of the assay to be performed in X-Vivo 15 working medium, (ii) plate block in X-Vivo 15 working medium containing 10 % AB serum for at least 1 h, (iii) restimulation of cells with peptide at 1 μg/mL final concentration and (iv) plating of 1 × 10^4^ cells per well (test viral antigens) or 1 × 10^5^ cells per well (TAA and viral control antigen). All reagents required for set up were supplied by the organising centre, with the exception of an appropriate wash medium (RPMI or equivalent). For both Phase I and II, protocols and reagents required for spot development were the centre’s own.

*Multimer Staining:* For both Phase I and II, all centres used their own established protocol for multimer staining, with some mandatory requirements: (i) a minimum of 1 × 10^6^ (ex vivo) or 0.5 × 10^6^ (post-IVS) cells per test were to be stained with all cells to be acquired, (ii) prior centrifugation of multimers at 13,000 rpm, 4 °C for 6 min, (iii) incubation of cells with multimer at a final concentration of 5 μg/mL for 30 min at room temperature and (iv) surface staining with anti-CD3, anti-CD8, anti-CD4 and anti-CD45RA antibodies as a minimum, with the option to include additional markers such as a dead cell marker, dump channel or other T-cell memory phenotype markers (Supplementary MIATA Information, Multimer Module 2). Centres supplied their own reagents throughout, with the exception of multimers.

### Harmonised in vitro stimulation

A harmonised IVS protocol was designed based upon features of the “best-performing” protocol(s) from Phase I, defined as one which delivered high antigen-specific T cells whilst maintaining low background and low inter-assay variation. The harmonised IVS protocol was then tested by 6 centres in Phase II. In brief, PBMCs were thawed, washed and resuspended in X-Vivo 15 medium (Lonza) supplemented with 1 % l-glutamine (200 mM; PAA), 1 % Penicillin/Streptamycin (10,000U/mL and 10,000mcg/mL; PAA) and 10 % human AB serum (Lonza). Cells were counted and volume adjusted to 4 × 10^6^ viable cells/mL. One mL of cell suspension was added to each well of a 24-well, flat-bottomed plate, along with 1 mL of X-Vivo 15 working medium plus antigenic peptides (Peptide Synthetics Peptide Protein Research Ltd.) and recombinant IL-2 (R&D Systems Europe Ltd.) to give a final concentration of 1 μg/mL and 20 IU/mL, respectively, in a final volume of 2 mL. Plates were incubated at 37 °C with 5 % CO_2_. On days 4, 6, 8 and 11, 1 mL of culture medium was removed from each well and replaced with 1 mL of X-Vivo 15 working medium plus recombinant IL-2 to a final concentration of 20 IU/mL. On day 13, the cells were harvested, washed twice and resuspended into X-Vivo 15 working medium. Cells were counted and volume adjusted to 1 × 10^6^ viable cells/mL for subsequent ELISPOT assay and multimer staining.

### Data acquisition

Each centre was required to complete a detailed questionnaire specifying the protocols and reagents used for the assays, including details of cell thawing, cell counting and QC of material, ex vivo ELISPOT and multimer staining, IVS and post-IVS ELISPOT and multimer staining.

Enumeration of IFNγ-producing cells was performed by participating centres using a suitable ELISPOT plate reader system and software. All raw data (SFC per well) were entered into a report form (Microsoft Excel spread sheet format) and supplied to the organising centre for centralised analysis.

For the analysis of multimer staining, samples were acquired by participating centres on a suitable flow cytometer. All raw data (FCS files) were returned to the organising centre for centralised gating using FlowJo software (Treestar Inc., Ashland, USA). All centres in Phase I and II, with the exception of Centre C, used a Live/Dead discrimination dye and therefore samples were firstly gated on a live cell population. A lymphocyte gate based on size and granularity followed, before defining CD3^+^CD4^−^CD8^+^multimer^+^ cells. As the donors were known to be HIV seronegative, HIV-multimer^+^ populations were used as a negative control and test antigen multimer^+^ gates were set accordingly.

### Data analysis

Centralised analysis of the raw data was performed by the organising centre. ELISPOT data were first processed by expressing each well as SFC per million PBMCs as per validation of ELISPOT assay [18, 19], followed by subtracting the mean spot number of the triplicate of unstimulated cells from that of the test triplicate. A mean and SD were calculated for each antigen-specific triplicate. An antigen-specific response was reported if the mean was both ≥20 (ex vivo) or ≥500 (post-IVS) SFC per million PBMCs and 2 SD above the mean of HIV stimulated wells; an informed threshold value for positivity above background (SFC/million) was applied for ex vivo and post-IVS ELISPOT based on previous data from the organising centre.

Multimer^+^ T cells were expressed as a percentage of the total CD3^+^CD4^−^CD8^+^ T cells analysed; a mean and SD were calculated for each antigen-specific multimer (from 3 repeated assays). All dot plots (test and control) were examined by 3 independent analysts in a blinded fashion and scores were given based upon both the frequency and appearance of multimer^+^ T cells- (2) a clustered population, (1) ambiguous population (0) clearly negative. A positive response was defined as a combined score of ≥5.

Inter-assay (for each centre) and inter-laboratory variability was calculated using the coefficients of variation (CV): % CV = SD/mean × 100. Correlations were evaluated using the Pearson’s correlation coefficient (R) and the coefficient of determination (*R* ^2^), with significance testing using Student’s *t* test and a confidence level of 99 %.

## Results

All centres completed the required assays and returned the raw data to the organising centre for central analysis, which included all replicates unless specifically stated (Supplementary MIATA Information, ELISPOT and Multimer Module 4A).

### Cell recovery and cell viability

To confirm that each centre was provided with PBMCs of comparable quality, cell recovery and cell viability were assessed after initial thawing (Supplementary Fig. 1, left panels). Cell recovery after thawing varied for each donor and for each centre, consistent with a non-standardised method of thawing, whilst cell viability was consistently high.

Cell recovery (relative to the cell number plated on day 1) and cell viability were also assessed after IVS (Supplementary Fig. 1, right panels). In Phase I, all participants, except Centre A, recovered fewer cells following IVS than were plated. No relationship was observed between the length of IVS and the cell recovery post-IVS; however, the cytokine milieu did affect the number of cells recovered (Supplementary Fig. 2). All 5 centres added IL-2 to the IVS, with 3 centres (A, B and C) using additional cytokines. The IVS protocol used by Centre A required the addition of IL-2, IL-15 and T-cell growth factor and displayed a significantly superior cell recovery for each of the 3 donors (mean 147 %, *P* < 0.0001.); Centres B and C used IL-2, IL-4 and IL-7 with a mean recovery of 61 % compared to 31 % for Centres D and E that used IL-2 alone. After harmonised IVS, cell recovery and cell viability were consistent across all centres (Supplementary Figs. 1 and 2).

### Phase I: Detection of antigen-specific T cells by ELISPOT and multimer staining, ex vivo compared to post-IVS

Phase I of the panel required that each of 5 participating centres analysed PBMCs from 3 donors (1–3) for the presence of T cells specific for HLA-A*0201-restricted epitopes from FLU and CMV, as well as HIV, which served as a negative control (Supplementary Table 1). Detection was by both ELISPOT and multimer staining, ex vivo and post-IVS. Pre-screening of donor PBMCs at the organising centre-identified donors as high (donors 1 and 2) and low (donor 3) for FLU-specific T cells, whilst CMV-specific responses were low/undetectable in all 3 donors.

**Fig. 2:**
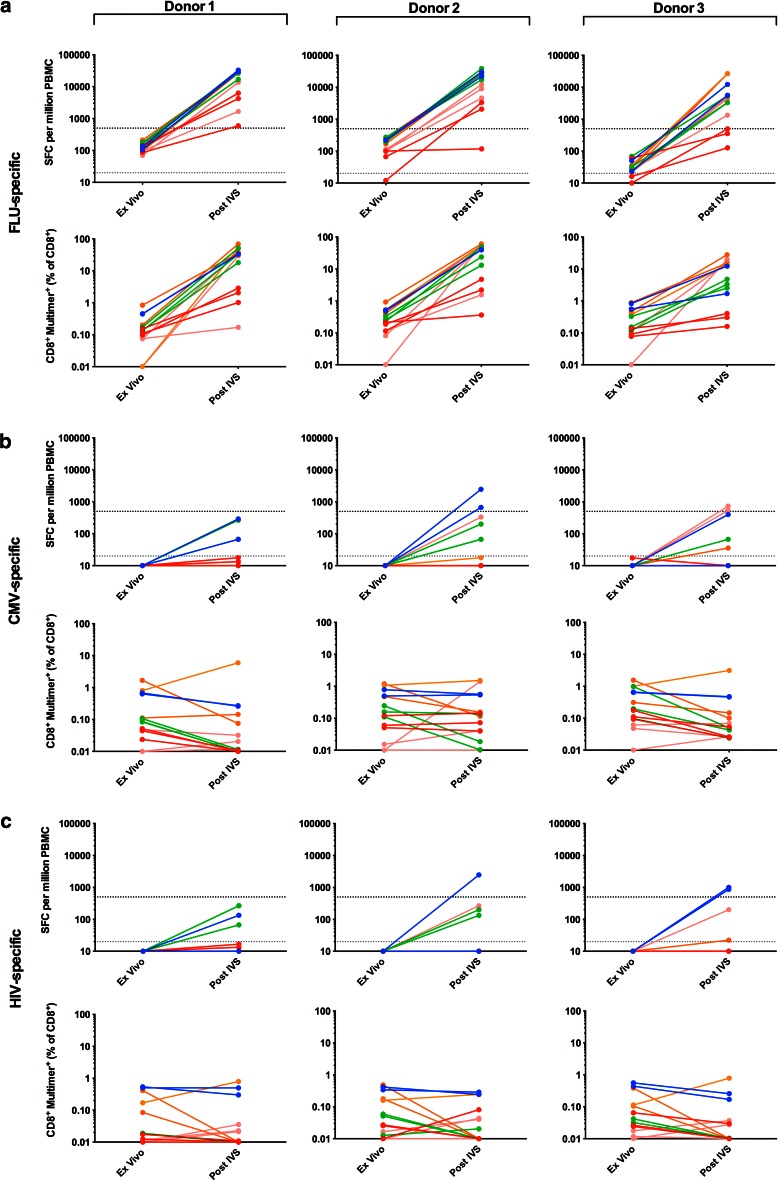
Antigen-specific T-cell responses observed ex vivo and post-IVS in Phase I The responses of 3 donors (1–3) to the viral antigens FLU (**a**), CMV (**b**) and HIV (**c**) were assessed by IFNγ ELISPOT assay and multimer staining, both ex vivo and post-IVS. The criteria for a positive response for ELISPOT and multimer are as described in the *Materials and Methods* and Supplementary MIATA Information, ELISPOT and Multimer Module 4B. Triplicates are shown for each centre except Centres A and E for which only two sets data were reported due to technical difficulties with one replicate; Centre A, post-IVS ELISPOT and multimer staining replicate 1; Centre E, ex vivo multimer staining replicate 3. Centre A, *blue*; Centre B, *red*; Centre C, *green*; Centre D, *orange*; Centre E, *pink*. *Grey dashed line* denotes the threshold for a positive response in ex vivo ELISPOT (20 SFC/million); *black dashed line* denotes the threshold for a positive response in post-IVS ELISPOT (500 SFC/million). For Centre D post-IVS ELISPOT (donor 1–3, replicate 1–3) test wells were saturated with SFC too numerous to count, therefore, these wells were reported with a maximum spot number of 2000. ND, not detected

All 5 centres detected FLU-specific T cells in each of the 3 donors by ex vivo ELISPOT and multimer staining (Fig. 2a; Table 1a). Mean FLU-specific responses were highest in donor 2 with 180 SFC/million (ELISPOT) and 0.352 % CD8^+^FLU-multimer^+^ T cells (multimer), compared to 132 SFC/million and 0.275 % CD8^+^FLU-multimer^+^ and 35 SFC/million and 0.299 % CD8^+^FLU-multimer^+^ for donors 1 and 3, respectively. Following IVS, all centres could detect FLU-specific T cells in all 3 donors by multimer staining, but only 4 centres (A, C-E) by ELISPOT (Fig. 2a; Table 1a); Centre B detected FLU^+^ T cells in donors 1 and 2, but not 3. Similar to ex vivo analysis, mean FLU-specific responses were greatest in donor 2 with 18729 SFC/million (ELISPOT) and 31.8 % CD8^+^FLU-multimer^+^ T cells (multimer), compared to 17627 SFC/million and 26.7 % CD8^+^FLU-multimer^+^ and 12865 SFC/million and 8.0 % CD8^+^FLU-multimer^+^ for donors 1 and 3, respectively. Overall, the detection rate for ex vivo multimer staining was 93 % increasing to 98 % post-IVS. However, for ELISPOT, this decreased from 91 % ex vivo to 86 % post-IVS, attributable to weak responses in donor 3. The IVS protocol used by Centre D consistently gave the best performance with the greatest detection rate, SFC/million count and % CD8^+^FLU-multimer^+^ T cells, indicative of a robust IVS protocol.

**Table 1:** Detection rates of antigen-specific responses assessed by ELISPOT and multimer staining: (a) Phase I, ex vivo and post-IVS and (b) Phase II, post-IVS alone

		Donors	Centre A			Centre B			Centre C			Centre D			Centre E			Mean (%)			
			1	2	3	1	2	3	1	2	3	1	2	3	1	2	3	1	2	3	
(a)																					
FLU^a^	ELISPOT	Ex vivo	3/3	3/3	3/3	3/3	2/3	1/3	3/3	3/3	3/3	3/3	3/3	2/3	3/3	3/3	3/3	100	93	80	91
		Post-IVS	2/2^c^	2/2^c^	2/2^c^	3/3	2/3	0/3	3/3	3/3	2/3	3/3	3/3	3/3	3/3	3/3	2/3	100	93	64	86
	Multimer	Ex vivo	3/3	3/3	1/3	3/3	3/3	3/3	3/3	3/3	3/3	2/2^d^	3/3	2/3	2/2^c^	2/2^c^	2/2^c^	100	100	79	93
		Post-IVS	2/2^c^	2/2^c^	2/2^c^	3/3	3/3	3/3	3/3	3/3	3/3	3/3	3/3	3/3	3/3	3/3	2/3	100	100	93	98
CMV^b^	ELISPOT	Ex vivo	0/3	0/3	0/3	0/3	0/3	0/3	0/3	0/3	0/3	0/3	0/3	0/3	0/3	0/3	0/3	0	0	0	0
		Post-IVS	0/2^c^	1/2^c^	0/2^c^	0/3	0/3	0/3	0/3	0/3	0/3	0/3	0/3	0/3	0/3	0/3	0/3	0	7	0	2
	Multimer	Ex vivo	0/3	2/3	0/3	0/3	0/3	0/3	1/3	0/3	0/3	0/3	2/3	0/3	0/2^c^	0/2^c^	0/2^c^	7	29	0	12
		Post-IVS	0/2^c^	0/2^c^	0/2^c^	0/3	0/3	0/3	0/3	1/3	2/3	1/3	2/3	1/3	0/3	0/3	0/3	7	20	20	17

	Donors	Centre A		Centre B		Centre C		Centre D		Centre E		Centre F		Mean (%)		
		4	5	4	5	4	5	4	5	4	5	4	5	4	5	
(b)																
EBV^e^	ELISPOT	3/3	3/3	3/3	3/3	3/3	3/3	3/3	2/3	3/3	3/3	3/3	3/3	100	94	97
	Multimer	3/3	3/3	2/2^d^	3/3	3/3	3/3	3/3	3/3	3/3	3/3	3/3	3/3	100	100	100
FLU^f^	ELISPOT	3/3	3/3	2/3	2/3	0/3	0/3	1/3	1/3	3/3	3/3	3/3	2/3	67	61	64
	Multimer	3/3	3/3	3/3	3/3	3/3	3/3	3/3	3/3	3/3	3/3	3/3	3/3	100	100	100
WT1 ^g^	ELISPOT	0/3	0/3	0/3	0/3	0/3	0/3	0/3	0/3	0/3	0/3	0/3	0/3	0	0	0
	Multimer	1/3	2/3	1/3	2/3	2/3	2/3	2/3	2/3	0/3	2/3	0/3	1/3	33	61	47

Antigen-specific response criteria, ELISPOT: ≥20 (ex vivo) or ≥500 (post-IVS) SFC/million PBMC and 2 SD above mean HIV control. Antigen-specific response criteria, multimer: combined visual examination score of dot plots ≥5

*ND* not detected

^a^Mean FLU-specific T cell detection rate for post-IVS ELISPOT and multimer combined was 100, 78, 94, 100 and 89 % for Centres A, B, C, D and E, respectively (donors 1, 2 and 3)

^b^Mean CMV-specific T cell detection rate for post-IVS ELISPOT and multimer combined was 8 %, ND, 17, 22 % and ND for Centres A, B, C, D and E, respectively (donors 1, 2 and 3)

^c^Only two sets of data were reported due to technical difficulties with one replicate

^d^Only two FCS files were supplied by the centre for centralised analysis

^e^Mean EBV-specific T cell detection rate for post-IVS ELISPOT and multimer combined was 100, 100, 100, 92, 100 and 100 % for Centres A, B, C, D, E and F, respectively (donors 4 and 5)

^f^Mean FLU-specific T cell detection rate for post-IVS ELISPOT and multimer combined was 100, 83, 50, 67, 100 and 92 % for Centres A, B, C, D, E and F, respectively (donors 4 and 5)

^g^Mean WT1-specific T cell detection rate for post-IVS multimer combined was 25, 25, 33, 33, 17 and 8 % for Centres A, B, C, D, E and F, respectively (donors 4 and 5)

**Table 2:** Summary of mean inter-assay and inter-laboratory % coefficients of variation for Phase I and Phase II: (a) high/intermediate/low response and (b) low/undetectable response

			ELISPOT: %CV		Multimer: %CV	
			Inter-assay	Inter-laboratory	Inter-assay	Inter-laboratory
(a)						
Phase I	Ex vivo	FLU	40.6	66.9	33.3	83.2
Phase I	Post-IVS	FLU	48.7	84.6	51.5	84.6
Phase II		EBV/FLU	55.9	70.6	48.5	58.5
		Mean	48.4 ± 7.7		44.4 ± 9.8 %	

			ELISPOT: %CV		Multimer: %CV	
			Inter-assay	Inter-laboratory	Inter-assay	Inter-laboratory
(b)						
Phase I	Ex vivo	CMV	126.0	163.2	49.1	115.0
Phase I	Post-IVS	CMV	127.9	217.2	76.1	239.8
Phase II		WT1	N/A	N/A	51.2	113.3
		Mean	127.0 ± 1.3		58.8 ± 15.0 %	

*N/A* not applicable

Three centres (A, C and D) detected CMV-specific responses in 1/3 donors by ex vivo multimer staining, with a mean of all positive responses of 0.776 % CD8^+^CMV-multimer^+^ T cells (Fig. 2b); a mean detection rate of 7 and 29 % was observed for donors 1 and 2, respectively (Table 1a). In contrast, ex vivo ELISPOT did not reveal CMV-specific T cells. Following IVS, Centres C and D continued to detect CMV^+^ T cells by multimer staining in up to 3 donors (mean 0.104 % CD8^+^CMV-multimer^+^); a mean detection rate of 7, 20 and 20 % was observed for donors 1, 2 and 3, respectively (Fig. 2b; Table 1a). Only Centre A detected CMV^+^ T cells by post-IVS ELISPOT, which was observed only in donor 2 in 1/2 replicates. Overall, the combined CMV detection rate was 6 % ex vivo and 10 % post-IVS.

No T-cell responses to HIV reached the threshold for a positive response based on the pre-define criteria (see *‘Materials and Methods, Data analysis’* and Supplementary MIATA Information, ELISPOT and Multimer Module 4B) and were thus deemed negligible (Fig. 2c), consistent with these blood donors being HIV negative.

Example raw data and mean SFC/million and % multimer^+^ T cells are shown in Supplementary MIATA Information, ELISPOT and Multimer Module 3B.

### Amplification of antigen-specific T-cell responses post-IVS

Amplification of FLU-specific T cells following IVS versus ex vivo assay was assessed in Phase I (Supplementary Table 2). The magnitude of T-cell amplification was highly variable depending on the donor PBMCs, IVS protocol and means of detection. In general, amplification was greatest when detection was via ELISPOT, with an overall mean fold increase of 185.1 ± 238.5 (ELISPOT) compared to 98.1 ± 142.9 (multimer).

### Background IFNγ production in ELISPOT, ex vivo and post-IVS

The impact of IVS on the background level of IFNγ production detected in ELISPOT was assessed by comparing the mean SFC/million count of unstimulated cells detected ex vivo with that post-IVS for each of 5 centres in Phase I (example raw data shown in Supplementary MIATA Information, ELISPOT Module 3B). Background IFNγ production increased following IVS for all centres; mean 9, 7 and 4 SFC/million ex vivo compared to 86, 254 and 135 SFC/million (mean from centres B-E only) post-IVS for donors 1, 2 and 3, respectively. Centre A displayed very high background IFNγ production following IVS; 1489, 2156 and 6711 SFC/million for donors 1, 2 and 3, respectively. A resting phase (~ 24 h) after IVS and prior to ELISPOT plate set up was included by Centre E; however, this did not appear to reduce background IFNγ production.

### Development of a harmonised IVS protocol

A harmonised IVS protocol was designed to incorporate features from the “best-performing” protocol(s) from Phase I, in order to produce a method capable of delivering high amplification of antigen-specific T cells with a maximum detection rate whilst displaying limited inter-assay variability. The IVS protocol used by Centre D resulted in 100 % detection rate of FLU-specific T cells (ELISPOT and multimer) and generated the greatest FLU-specific T-cell responses (mean 26615 SFC/million and 42.6 % CD8^+^FLU-multimer^+^ T cells). Moreover, it displayed the largest (ELISPOT) and third largest (multimer) fold increase in FLU-specific T cells compared to ex vivo and the lowest inter-assay variation for multimer (see *‘Phase I/II: Inter*-*assay and inter*-*laboratory variation’*; not evaluable for ELISPOT), whilst maintaining a low background. Therefore, the harmonised protocol designed (see *‘Materials and Methods, Harmonised* in vitro *stimulation’*) was most similar to that used by Centre D; however, some modifications to the protocol were incorporated for ease of multi-centre application.

### Phase II: Detection of antigen-specific T cells by ELISPOT and multimer staining post-harmonised IVS

Phase II of the panel required that each of 6 participating centres analyse PBMCs from 2 donors (4–5) for the presence of CD8^+^ T cells specific for HLA-A*0201-restricted epitopes from FLU, EBV and the TAA Wilm’s tumour antigen 1 (WT1), as well as HIV, which served as a negative control (Supplementary Table 1). Detection was by both ELISPOT and multimer staining following harmonised IVS. Pre-screening of donor PBMCs at the organising centre-identified donors as high (donor 4) and intermediate (donor 5) for EBV-specific T cells, low (donors 4 and 5) for FLU-specific T cells and low/undetectable (donor 5 alone) for WT1-specific T cells.

**Fig. 3:**
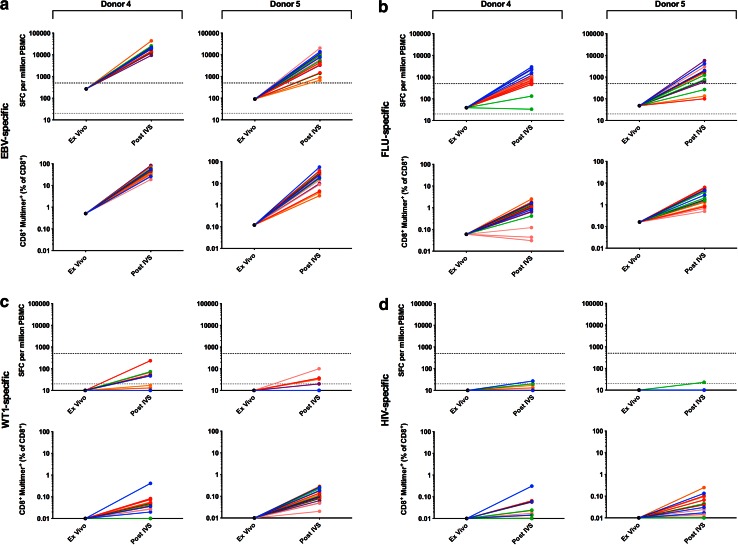
Antigen-specific T-cell responses observed post-harmonised IVS in Phase II The responses of 2 donors (4 and 5) to the viral and tumour-associated antigens EBV (**a**), FLU (**b**), WT1 (**c**) and HIV (**d**) were assessed by IFNγ ELISPOT assay and multimer staining post-harmonised IVS; ex vivo data (mean of triplicate) generated by the organising centre during pre-screening are also shown. The criteria for a positive response for ELISPOT and multimer staining are as described in the *Materials and Methods* and Supplementary MIATA Information, ELISPOT and Multimer Module 4B. Triplicates are shown for each centre except Centre B (post-IVS multimer staining donor 4) for which only two FCS files were supplied to the organising centre for central analysis. Centre A, *blue*; Centre B, *red*; Centre C, *green*; Centre D, *orange*; Centre E, *pink*; Centre F, purple. *Grey dashed line* denotes the threshold for a positive response in ex vivo ELISPOT (20 SFC/million); *black dashed line* denotes the threshold for a positive response in post-IVS ELISPOT (500 SFC/million). ND, not detected

All 6 centres detected EBV-specific T cells in both donors by post-IVS ELISPOT and multimer staining (Fig. 3a); a mean detection rate of 100 and 94 % by ELISPOT and 100 and 100 % by multimer staining was observed for donors 4 and 5, respectively (Table 1b). Mean EBV-specific responses were greatest in donor 4 with 18813 SFC/million (ELISPOT) and 48.8 % CD8^+^EBV-multimer^+^ T cells (multimer), compared to 7445 SFC/million and 22.1 % CD8^+^EBV-multimer^+^ for donor 5. Overall, a combined detection rate of 97 and 100 % was observed for ELISPOT and multimer staining, respectively.

All 6 centres could detect FLU-specific T cells in both donors by post-IVS multimer staining, but only 5 centres (A, C–F) by ELISPOT (Fig. 3b); Centre C did not detect FLU^+^ T cells in either donor by ELISPOT. The mean detection rate was 67 and 61 % by ELISPOT and 100 and 100 % by multimer staining for donors 4 and 5, respectively (Table 1b). Mean FLU-specific responses were greatest in donor 5 with 2461 SFC/million (ELISPOT) and 3.0 % CD8^+^FLU-multimer^+^ T cells (multimer), compared to 1314 SFC/million and 1.0 % CD8^+^FLU-multimer^+^ for donor 4. Overall, a combined detection rate of 64 and 100 % was observed for ELISPOT and multimer staining, respectively.

All 6 centres could detect low levels of WT1-specific T cells in one or both donors by post-IVS multimer staining (Fig. 3c); a mean detection rate of 33 and 61 % was observed for donors 4 and 5, respectively (Table 1b). Mean WT1-specific responses were greatest in donor 5 with 0.12 % CD8^+^WT1-multimer^+^ T cells compared to 0.04 % for donor 4. In contrast, no centre was able to detect WT1-specific T cells by post-IVS ELISPOT.

No T-cell responses to HIV reached the threshold for a positive response based on the pre-define criteria (see *‘Materials and Methods, Data analysis’* and Supplementary MIATA Information, ELISPOT and Multimer Module 4B) and were thus deemed negligible (Fig. 3d), consistent with these blood donors being HIV negative.

Example raw data and mean SFC/million and % multimer^+^ T cells are shown in Supplementary MIATA Information, ELISPOT and Multimer Module 3B.

### Background IFNγ production in ELISPOT post-harmonised IVS

The mean background IFNγ production after harmonised IVS in Phase II was comparable to that observed in Phase I; 341 and 65 SFC/million for donors 4 and 5, respectively (example raw data is shown in Supplementary MIATA Information, ELISPOT Module 3B).

### Phase I/II: Inter-assay and inter-laboratory variation

Inter-assay (for each centre) and inter-laboratory %CVs were calculated for Phase I and II data, as summarised in Table 2. The inter-assay variation of ELISPOT and multimer assays remained consistent whether measured ex vivo or post-IVS as would be predicted based on the execution of replicates by a common operator (Table 2; for a breakdown of the inter-assay %CV for individual centres see Supplementary Tables 3 and 4). A lower level of inter-assay variation was observed for responses that were robustly detected (high/intermediate/low response) compared to low/undetectable responses; mean combined inter-assay %CV was 48.4 ± 7.7 % and 44.4 ± 9.8 % (high/intermediate/low response) and 127.0 ± 1.3 and 58.8 ± 15.0 % (low/undetectable response) for ELISPOT and multimer staining, respectively. In Phase I, IVS of PBMCs prior to assay by ELISPOT and, to a lesser extent multimer staining, resulted in an increase in the mean inter-laboratory %CV compared to ex vivo assay (Table 2 and Supplementary Table 5); an increase by 17.7 and 1.4 % (high/intermediate/low response) and 54.0 and 124.8 % (low/undetectable response) for ELISPOT and multimer staining, respectively. In Phase II, which used a harmonised IVS protocol that included the use of centrally prepared reagents, the mean inter-laboratory %CV was reduced (Table 2 and Supplementary Table 6). For robust responses (high/intermediate/low response), inter-laboratory %CV decreased by 14.0 and 26.1 % for ELISPOT and multimer staining, respectively, whilst for low/undetectable responses a decrease of 126.5 % was observed for multimer staining; inter-laboratory %CV could be calculated for ELISPOT since no responses were detected.

### Phase I/II: Correlation of ex vivo, post-IVS, ELISPOT and multimer responses

**Fig. 4:**
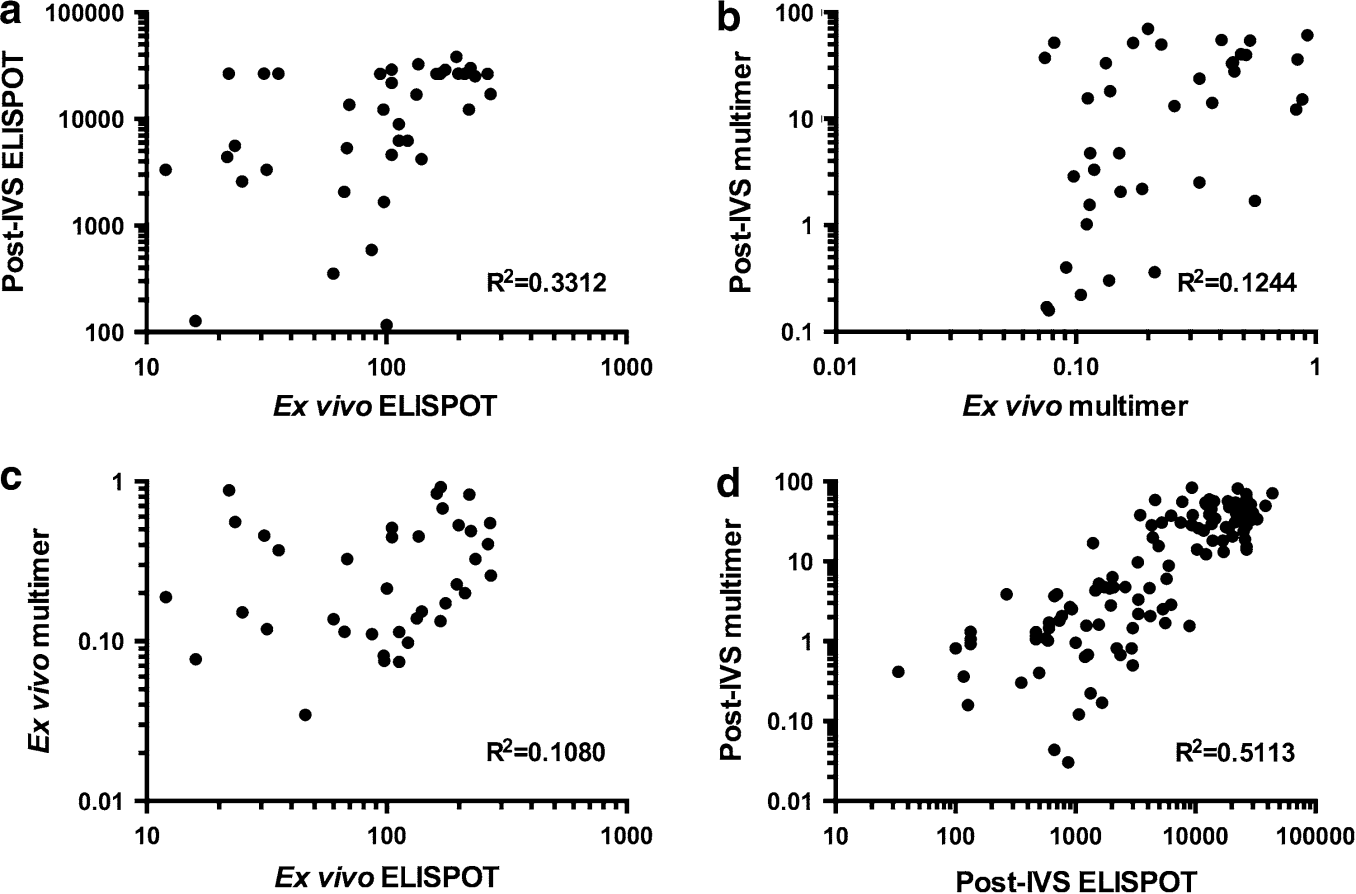
Correlation of ex vivo and post-IVS and ELISPOT and multimer responses in Phase I and II The responses of all donors (1–5), where appropriate, were assessed for the existence of significant (<0.01 %) correlation: Phase I, ex vivo ELISPOT with post-IVS ELISPOT (**a**); ex vivo multimer with post-IVS multimer (**b**); ex vivo ELISPOT with ex vivo multimer (**c**); Phase I and II, post-IVS ELISPOT with post-IVS multimer (**d**). Correlations were evaluated using the Pearson’s correlation coefficient (*R*) and the coefficient of determination (*R* ^2^), as shown on the bottom right of each graph. Significance testing used the Student’s *t* test and a confidence level of 99 %

Phase I ex vivo and post-IVS responses detected by both ELISPOT and multimer staining were evaluated for the existence of any correlation using *R* ^2^ and significance testing at 99 % confidence (Fig. 4 and Supplementary Table 7). Overall, a significant (*P* < 0.0001) positive correlation was observed between ex vivo ELISPOT and post-IVS ELISPOT, but was associated with a weak *R* ^2^ value of 0.3312 (Fig. 4a). Upon dissection and examination at the level of individual donors, significance of this correlation was only maintained for donor 2 (*R* ^2^ = 0.5411 and *P* = 0.0027). There was no correlation observed between ex vivo multimer and post-IVS multimer or between ex vivo ELISPOT and ex vivo multimer (Fig. 4b, c). Examination of Phase I and II post-IVS ELISPOT and multimer responses revealed a significant (*P* < 0.0001) positive correlation with an *R* ^2^ value of 0.5113 (Fig. 4d).

## Discussion

Data generated from this multi-centre exploratory panel have enabled a better understanding of the impact of IVS on the detection of antigen-specific T cells, including an insight into performance characteristics of different IVS protocols. In Phase I, we compared 5 different IVS techniques, and despite the large number of variables between protocols, all centres were able to expand and detect antigen-specific T cells above a pre-defined criteria for positivity. FLU-specific T cells were found in all three donors, but at varying frequencies. The two donors that displayed the highest frequencies ex vivo also had the highest frequencies following IVS, and in parallel the greatest detection rate (often 100 %). The third donor displayed a lower frequency of FLU-specific T cells and this corresponded to a reduced rate of detection, both for ex vivo and post-IVS assay. This was more pronounced for ELISPOT where ‘missed’ responses were in the main due to the number of IFNγ SFC falling short of 2 SD above background (HIV stimulated wells), and thus failing to achieve the criteria for a positive response. We had made recommendations to participating centres on the number of cells per well to plate following IVS based on our own experience of the responses generated by these donors during pre-screening assays. In hindsight, this recommendation was not optimal since the degree of T-cell expansion achieved by each of the different IVS protocols varied greatly. For some centres, T-cell expansion after IVS was lower and, therefore, the detection of an antigen-specific T-cell response may have benefitted from plating more cells during assay set up. In situations where the presence and level of reactivities are unknown, it may also be beneficial to plate cells at two concentrations to increase accurate enumeration of the antigen-specific T-cell frequency. We identified this also as the cause for responses missed by multimer staining, where too few CD8^+^ T cells were acquired (below 100,000 events). This factor has already been identified and reported as key for detecting antigen-specific T cells by multimer [10, 11].

A harmonised IVS protocol was designed based on the data from Phase I for further testing. Both donors investigated in Phase II had high/intermediate frequencies for EBV- and low for FLU-specific T cells ex vivo (pre-screening), and these were expanded well and reliably across all 6 centres using the harmonised IVS protocol. High detection rates were achieved, with consistent 100 % detection by multimer staining. As with Phase I, positive responses ‘missed’ by ELISPOT were associated with a lower frequency of antigen-specific T cells (FLU), which may be attributed to plating too few cells at set up. Overall, the higher the ex vivo frequency of the response the less variability in expansion was observed across the centres. As expected, the use of the harmonised IVS protocol reduced inter-laboratory variation.

Examination of the T-cell reactivities of our donors by both ELISPOT and multimer staining has enabled us to interpret the ex vivo and post-IVS assays with more clarity. In Phase I, FLU-specific T cell responses detected by ELISPOT ex vivo compared to post-IVS showed a correlation (*P* < 0.0001), but with a low *R* ^2^ value. Furthermore, when these responses were dissected and examined for individual donors, significance in this correlation was maintained for only 1/3 donors tested. The correlation was therefore donor dependent and must be a reflection of the phenotype of the cells present in vivo at the time samples were taken. Other studies in HIV [13], EBV [20], Hepatitis C [21] and malaria [22] have found that there is no correlation between ex vivo and cultured ELISPOT when comparing T-cell frequencies to the same antigen. These studies compared groups of donors and did not dissect the data at the level of the individual. Furthermore, our data show that ex vivo ELISPOT and multimer did not correlate and illustrate that these assays measure different T-cell populations within a heterogeneous population of antigen-specific T cells.

However, post-IVS, our data from Phase I and II combined demonstrated that a significant correlation became visible between T cell responses detected by ELISPOT and multimer staining (*P* < 0.0001 and *R* ^2^ = 0.5113). A likely explanation is that antigen-specific memory T cells proliferate and acquire an effector phenotype that can readily produce IFNγ for detection by both ELISPOT and multimer staining during IVS [23]. This is also supported by a study by Todryk et al. [12] that showed diminished IFNγ antigen-specific T-cell responses post-IVS when central memory cells are depleted pre-culture.

An important aspect of this study concerned the confidence by which a low level, borderline response can be affirmed to be a true positive or a true negative, a common quandary when testing for T-cell reactivities of unknown but anticipated low magnitude in immunotherapy clinical trials. In the past, we have unambiguously detected PSMA-specific T cells in vaccinated patients following, but not before, IVS by ELISPOT, suggesting that IVS can indeed be a useful tool for revealing reactivities that are undetectable or ambiguous ex vivo [15, 24]. However, in this study, the ambiguous CMV and WT1 responses were not resolved either ex vivo or following IVS despite a large number of assays performed within as well as across centres and by two methods. An increase in the number of assay replicates is generally beneficial in confirming a result; however, where there is inconsistency, as in this study, the decision making process is not helped. With limited patient samples available in trials, a large number of assay repeats may further not be feasible. In these cases, a complementary assay to confirm the presence or absence of antigen-specific response may instead be a viable alternative to assay repeats.

However, care must be taken not to disregard a positive response should the result from multiple assays be conflicting, particularly if the assays measure different T cell qualities such as structural presence of an antigen-specific TCR (multimer) as opposed to a functional ability of antigen-specific T cells (ELISPOT). There are two extra considerations that may lend weight to a clear response in the multimer assay. In contrast to the ELISPOT, the number of cells evaluated can be increased during the experiment, limited only by the total number of cells available. Therefore, the ability to detect low frequency T-cell responses ex vivo may be improved by multimer analysis over ELISPOT. Additionally, multimer staining captures T cells independently of the cell’s functional characteristics and is not influenced by the presence of immunosuppressive cells that may also circulate in patients and that will also be included in the test assay.

Our overall conclusion is that IVS can be a robust and reproducible tool that can be applied even in the multi-centre setting. A several log fold expansion of cells is achievable and that opens extra opportunity for further study, for example, for TCR rescue and cloning. Clearly IVS is not the final solution in every case to resolve the ambiguity of a borderline T-cell reactivity measured ex vivo as we demonstrate here. The most robust discrimination appears to result from a combination of a structural and functional evaluation in parallel. This is the approach we are taking for our future studies by building sample collection in a way that takes this into account.

## Electronic supplementary material

Below is the link to the electronic supplementary material.

Supplementary material 1 (PDF 411 kb)

## Abbreviations

CIC-CRI: Cancer Immunotherapy Consortium of the Cancer Research Institute
CIMT: The Association for Cancer Immunotherapy
CIP: CIMT Immunoguiding Program
CV: Coefficient of variation
IVS: In vitro stimulation
MIATA: Minimal information about T cell assays
SFC: Spot forming cells
TAA: Tumour associated antigen
WT1: Wilm’s tumour antigen 1
