# Supplementary material for: Harmonisation of short-term in vitro culture for the expansion of antigen-specific CD8+ T cells with detection by ELISPOT and HLA-multimer staining
Source: Cancer Immunol Immunother. 2014 Aug 19;63(11):1199–211. doi: 10.1007/s00262-014-1593-0 (PMC4209099; doi:10.1007/s00262-014-1593-0)
Supplement: Supplementary file 1 — Supplementary material 1 (PDF 411 kb) [file 262_2014_1593_MOESM1_ESM.pdf]

Cancer Immunology, Immunotherapy (submitted in 2014)- Lindsey Chudley *et al*.

Supplementary MIATA Information

**Module 1 – Sample PHASE I/II**		**Prepared in central laboratory**					
**1A – Donor**	Essential donor information	5 HLA-A2+ healthy volunteers (3 used in Phase I, 2 in Phase II)					
**1B – Source**	Source	National Blood Service					
	Collection method	PBMCs were isolated from anonymised buffy cones (HIV status negative) obtained from the National Blood Service, Southampton University Hospitals NHS Foundation Trust.					
	Processing methodology	Ficoll density gradient separation, washed twice in RPMI 1640 + 1mM sodium pyruvate, 1% PSG (Invitrogen Ltd, Paisley, UK). PBMC were counted and stored in 1mL aliquots at 1-2x10^7/vial depending on total cell number.					
**1C – Cryopreservation and storage**	Fresh/Cryo?	Cryopreserved					
	Freezing process	PBMC aliquots were stored in liquid nitrogen until shipment on dry ice to participating centres, where they were returned to liquid nitrogen until required.					
	Medium used for freezing	RPMI + P/S + sodium pyruvate + 50% human AB serum, 10% DMSO					
**1D – Cell counting**	**Mean yield and viability**	**Centre A**	**Centre B**	**Centre C**	**Centre D**	**Centre E**	**Centre F**
	Cell recovery after thawing (Phase I, Donors 1-3) Mean; range	78.0; 60.5-101	40.6; 30-49	56.7; 36.8-90.5	51.1; 31.7-73	64.9; 42-87	N/A
	Cell viability after thawing (Phase I Donors 1-3) Mean; range	67.4; 58.4-72.9	90; 87-91	94.6; 83.3-100	80.2; 75.4-84.2	96.8; 93-99	N/A
	Cell recovery after thawing (Phase II Donors 4-5) Mean; range	77.0; 60-85.5	57.7; 38-65	61.3; 50.1-72.7	64.4; 56.2-70.9	94.5; 78-109	76; 61-97
	Cell viability after thawing (Phase II Donors 4-5)	76.6; 68.2-86.1	88.9; 88-89.7	86.6; 77.3-93	84.1; 79.9-86.8	92.8; 87-94	96.3; 94-99

	Mean; range						
	Cell counting method	Trypan Blue and haemocytometer	Guava Counter (ViaCount)	Trypan Blue and haemocytometer	Guava Cell Counter PCA-96	Trypan Blue and haemocytometer	Trypan Blue and haemocytometer
**Module 2 – Assay PHASE I IVS protocol**		**Centre A**	**Centre B**	**Centre C**	**Centre D**	**Centre E**	**Centre F**
	Resting phase	No	Overnight	Overnight	No	4 hours	N/A
	Cell number (per well)	1-2x10^6	5x10^6	5x10^6	4-5x10^6	2x10^6	N/A
	Well volume	1ml	2ml	Up to 2ml	2ml	2ml	N/A
	Plate	24-well	24-well	24-well	24-well	12-well	N/A
	Medium; source	IMDM; Lonza	IMDM; Invitrogen	IMDM; Lonza	X Vivo 15; Lonza	X Vivo 15; Lonza	N/A
	% human AB serum; source	10%; PAA	10%; Lonza	10%; Lonza	10%; PAN Biotech	5%; Lonza	N/A
	Peptide stimulation	Single	Pool	Pool	Pool	Single	N/A
	Peptide concentration	5µg/ml	1µg/ml	1µg/ml	1µg/ml	10µg/ml	N/A
	Feeder cytokines; concentration	IL-2, 5ng/mL<br>IL-15, 100IU/mL<br>TCGF (Gentaur), 10%	IL-2, 2ng/mL<br>IL-4, 5ng/mL<br>IL-7, 5ng/mL	IL-2, 2ng/mL<br>IL-4, 5ng/mL<br>IL-7, 5ng/mL	IL-2, 20IU/mL	IL-2, 20IU/mL	N/A
	Length of IVS (days)	7	12	13	13	9	N/A
**Module 2 – Assay PHASE II Harmonised IVS protocol**		**Same protocol used by every centre (Centres A-F)**					
	Resting phase	No					
	Cell number (per well)	4-5x10^6					
	Well volume	2ml					
	Plate	24-well					
	Medium; source	X Vivo 15; Lonza					
	% human AB serum; source	10%; Lonza					
	Peptide stimulation	Pool					

	Peptide concentration	1µg/ml					
	Feeder cytokines; concentration	IL-2, 20IU/mL					
	Length of IVS (days)	13					
**Module 2 – Assay PHASE I/II Ex vivo and post-IVS ELISPOT**		**Centre A**	**Centre B**	**Centre C**	**Centre D**	**Centre E**	**Centre F**
**2A – Medium/serum**	Medium/(serum) details	IMDM + 10%hAB (PAA) w/o DNAse	OpTmizer CTS T cell expansion SFM + supplement (Invitrogen A10485-01)	IMDM (Lonza BE12-722F) +P/S +50uM bME +10% HS	X-Vivo (Lonza) +10% hAB serum (PAN), Glutamine (Gibco), P/S (Lonza)	RPMI (Gibco/PAA) + 10%hAB serum (Lonza) +P/S/G	X-vivo 15 (Lonza) +1% P/S (PAA), 1% L-Glutamine (PAA), 10% hAB sérum (Lonza)
**2B – Assay**	Resting phase? Ex vivo (Phase I)	o/n	2h	o/n	22hrs	4hrs	N/A
	Resting phase? Post-IVS<br>Phase I<br>Phase II	No<br>No	No<br>No	No<br>No	No<br>No	Yes<br>No	N/A<br>No
	Number of cells per well ex vivo (Phase I)	400,000	100,000 or 250,000	400,000	400,000	400,000	N/A
	Number of cells per well post-IVS (Phase I)	5,000	150,000 /100,000	5,000 /25,000	75,000 (+75,000 K562-A2)	5,000	N/A
	Number of cells per well post-IVS (Phase II)	10,000 or 100,000	10,000 or 100,000	10,000 or 100,000	10,000 or 100,000	10,000 or 100,000	10,000 or 100,000
	Concentration of peptide (Phase I/II)	I µg/mL	I µg/mL	I µg/mL	I µg/mL	I µg/mL	I µg/mL
	How long were cells cultured?	18-20hrs	18hr	26hrs	22hrs	16-20hrs	16hrs
	Plate type	Nitrocellulose backed	Multiscreen HTS-HA filter	Multiscreen HTS HA opaque	96 well plate BD Biosciences	Multiscreen-IP 96 separation	Multiscreen filter plates

		multiscreen 96-well plates (Millipore MSHA S4510)	plate (Millipore, MAHAS4510)	plates (Millipore MSHAN4B50)	(551849)	system, (Millipore MAIPS4510)	(Millipore, MAHAS4510)
	Coating Ab	Anti-human IFN gamma Mab-1-D1K (Mabtech 3420-3-1000)	Anti-human IFN gamma; Klon Mab 1-D1K; 1mg/mL (Mabtech 3420-3-1000)	Anti-human IFN gamma Mab-1-D1K (Mabtech 3420-3-1000)	Purified anti-human IFN gamma (BD 51-2555KC)	Anti-human IFN gamma Mab-1-D1K (Mabtech 3420-3-1000)	Kit from DIACLONE 856.051.020
	Detecting Ab	Anti-human IFN gamma Mab-7-B6-1 biotinylated (Mabtech, 3420-6-1000)	Anti-human IFN gamma Mab-7-B6-1 biotinylated (Mabtech, 3420-6-1000)	Anti-human IFN gamma Mab-7-B6-1 biotinylated (Mabtech, 3420-6-1000)	Anti-human IFN gamma biotinylated (BD 51-1890KC)	Anti-human IFN gamma Mab-7-B6-1 biotinylated (Mabtech, 3420-6-1000)	Kit from DIACLONE 856.051.020
	Detecting reagent	Extravidin-ALP (Sigma E-2636) 1:1000 diluted in PBS BCIP/NBT-Alkaline phosphatase substrate (Sigma B-5655)	SigmaFAST BCIP/NBT (Sigma B-5655)	Extravidin-ALP (Sigma E-2636) 1:1000 diluted in PBS BCIP/NBT-Alkaline phosphatase substrate (Sigma B-5655)	Streptavidin-HRP (BD 51-9000209) AEC substrate set (BD 551951)	Streptavidin-ALP (Mabtech) BCIP/NBT-Alkaline phosphatase substrate (Zymed)	Kit from DIACLONE 856.051.020 BCIP/NBT
**2C – Controls**	Internal assay controls	Media only, HIV peptide control and PHA/SEB					
**Module 3 – Data acquisition (ELISPOT)**		**Centre A**	**Centre B**	**Centre C**	**Centre D**	**Centre E**	**Centre F**
**3A – Equipment and software**	ELISPOT reader	BioSys 5000 from Bio-Sys company, Karben, Germany	CTL ImmunoSpot Analyzer serial number: 029038	CTL Europe GmbH; Immunospot® Series 5 Core Analyzer	CTL ImmunoSpot® Series 3B Analyzer	A.I.D. ELISPOT Plate Reader ELR04	Bioreader 5000 BioSys
	ELISPOT software	Bioreader 10	CTL Immunospot 5.0 Pro DC	Immunocapture 6.1, Immunospot® Analysis software 4.0	CTL Immunospot Software	A.I.D. ELISPOT Software	Bioreader 10.8

**3B – Acquisition strategy and gating**	Strategy for establishment of spot detecting parameters	Each centre used established parameters for detecting spots tailored to the specific reader, software design and algorithms.					
	Representative data set: Post-IVS ELISPOT Phase II Donor 5	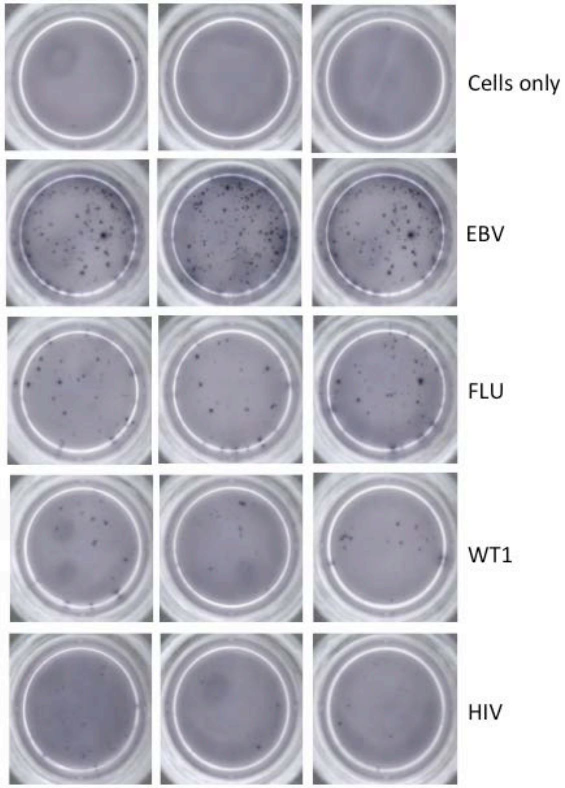					
	Mean IFN-γ SFC/million Donors 1-3 (I) Donors 4-5 (II)	**Centre A**	**Centre B**	**Centre C**	**Centre D**	**Centre E**	**Centre F**
	**Phase I** – FLU response	Ex vivo: 138<br>Post-IVS: 21933	Ex vivo: 96<br>Post-IVS: 288	Ex vivo: 145<br>Post-IVS: 20225	Ex vivo: 144<br>Post-IVS: 26615	Ex vivo: 74<br>Post-IVS: 7083	Ex vivo: N/A<br>Post-IVS: N/A
	**Phase I** – CMV response	Ex vivo: ND<br>Post-IVS: 2467	Ex vivo: ND<br>Post-IVS: ND	Ex vivo: ND<br>Post-IVS: ND	Ex vivo: ND<br>Post-IVS: ND	Ex vivo: ND<br>Post-IVS: ND	Ex vivo: N/A<br>Post-IVS: N/A
	**Phase I** – HIV response	Ex vivo: ND<br>Post-IVS: ND	Ex vivo: ND<br>Post-IVS: ND	Ex vivo: ND<br>Post-IVS: ND	Ex vivo: ND<br>Post-IVS: ND	Ex vivo: ND<br>Post-IVS: ND	Ex vivo: N/A<br>Post-IVS: N/A

	**Phase II** – EBV response	Ex vivo: N/A Post-IVS:15406	Ex vivo: N/A Post -VS: 472	Ex vivo: N/A Post-IVS: 2228	Ex vivo: N/A Post-IVS: 16527	Ex vivo: N/A Post-IVS: 16945	Ex vivo: N/A Post-IVS: 10711
	**Phase II** – FLU response	Ex vivo: N/A Post-IVS: 2456	Ex vivo: N/A Post-IVS: 1267	Ex vivo: N/A Post-IVS: ND	Ex vivo: N/A Post-IVS: 1084	Ex vivo: N/A Post-IVS: 1645	Ex vivo: N/A Post-IVS: 2200
	**Phase II** – WT1 response	Ex vivo: N/A Post-IVS: ND	Ex vivo: N/A Post-IVS: ND	Ex vivo: N/A Post-IVS: ND	Ex vivo: N/A Post-IVS: ND	Ex vivo: N/A Post-IVS: ND	Ex vivo: N/A Post-IVS: ND
	**Phase II** – HIV response	Ex vivo: N/A Post-IVS: ND	Ex vivo: N/A Post-IVS: ND	Ex vivo: N/A Post-IVS: ND	Ex vivo: N/A Post-IVS:ND	Ex vivo: N/A Post-IVS: ND	Ex vivo: N/A Post-IVS: ND
**Module 4 – Results (ELISPOT)**		**Centre A**	**Centre B**	**Centre C**	**Centre D**	**Centre E**	**Centre F**
**4A – Raw data**	Cut off specifications and # of tests OOS	Phase I, 1 replicate post-IVS ELISPOT all donors	N/A	N/A	N/A	N/A	N/A
	Accessibility of raw data addressed?	Raw data is held at each centre and analysed data can be made available upon request.					
**4B – Response determination**	Definition of positive reactivity (above background) incl. tests applied	An antigen-specific response was reported if the mean was both ≥20 (ex vivo) or ≥500 (post-IVS) SFC per million PBMCs and 2 SD above the mean of HIV stimulated wells. HIV-specific responses were reported if the mean was ≥20 (ex vivo) or ≥500 (post-IVS) SFC per million PBMCs.					
	Response definition pre-defined or post-hoc	Pre-defined					
**Module 2 – Assay PHASE I/II Ex vivo and post-IVS Multimer staining**		**Centre A**	**Centre B**	**Centre C**	**Centre D**	**Centre E**	**Centre F**
**2A – Medium/serum**	Wash/staining buffers	PBS + 0.5% BSA	Washing and surface staining: PBS +0.01% NaAzide + 1% EDTA +5% FCS Tetramner staining buffer: PBS +0.01%	Washing and surface staining: PBS +0.01% NaAzide +2mM EDTA +2% FCS Tetramer staining buffer: PBS +0.01% NaAzide +2mM EDTA +50%	PBS for Live Dead Aqua; PFEA (2% FCS, 0.4% EDTA and 0.1% natrium acid in PBS) for surface Ab	Washing and surface staining: PBS +0.01% NaAzide +0.5% BSA Tetramer staining buffer: PBS +0.1% NaAzide +50% FCS	PBS + 0.2% BSA +5mM EDTA +0.1% Sodium azide

			NaAzide + 2% EDTA +50% FCS	FCS			
**2B – Assay**	Cells rested before staining?	No	No	No	No	No	No
	Staining volume	100µl	50µl	50µl	NK	50µl	NK
	MHC multimer concentration	5µg/mL	5µg/mL	5µg/mL	5µg/mL	5µg/mL	5µg/mL
	Incubation conditions	Tetramer: 30 mins RT, dark Surface: 20 mins 4-8 C, dark	Tetramer: 30 mins RT, dark Surface: 20 mins 4-8 C, dark	Tetramer: 30 mins RT, dark Surface: 20 mins 4-8 C, dark	Tetramer: 30 mins RT, dark Surface: 30 mins 4-8 C, dark	Tetramer: 30 mins RT, dark Surface:15 mins 4-8 C, dark	Tetramer: 30 mins RT, dark Surface:15 mins 4-8 C, dark
	mAbs Fluorochrome; Company (cat number); clone						
	CD3	PB; Dako (PB982) UCHT1	PB; BD (558117); UCHT1	APC; Caltag; S4.1	APC-H7; BD (641397) ;SK7	HV450; BD (560365); UCHT1	APC-Alexa-Fluor 750; BC (A94680); UCH-T1
	CD8	APC; Dako (C7227); DK25	PE Cy7; BC (737661); SFCI21Thy2D3	PE-Cy7; Beckman Coulter; SFCI21Thy2D3	Qdot 705; Invitrogen (Q10059); 3B5	PerCP Cy5.5; BD (560662); RPA-T8	APC ; BC (IM2469) ; B9.11
	CD4	PE-Cy7; BD (557852) SK3	APC Cy7; BD (341115); SK3	APC-Cy7; BD; RPA-T4	Qdot 605; Invitrogen; S3.5	APC-H7; BD (641398); SK3	PB; BC (A82789); 13B8.2
	Dead cell dye	Propidium Iodide	7-AAD; BC (A07704)	No	Aqua; Invitrogen; (L34957)	Aqua; Invitrogen (L34957)	Vivid Aqua; Invitrogen (L34957)
	Dump channel?	No	No	No	No	No	No
	Cells fixed?	No	No	Yes. 1% formaldehyde	Yes. 1% formaldehyde	No	No
**2C – Controls**	Internal assay controls	HIV-multimer used as a negative control					
**Module 3 – Data acquisition (Multimer staining)**		**Centre A**	**Centre B**	**Centre C**	**Centre D**	**Centre E**	**Centre F**

**3A – Equipment and software**	Flow cytometer and software for acqusition	BD LSR Fortessa; BD FACS DIVA 6.2	BD FACS Canto II serial number: V96301151; Diva 6.1.3	BD FACS Canto II;<br>Diva 6.1.2	BD LSRII; DIVA 6.1.2	BD FACS Canto II; Diva 6.1.2	Gallios flow cytometer; Gallios Software
	Software for analysis	FlowJo software (Treestar Inc., Ashland, USA.)					
**3B – Acquisition strategy and gating**		Samples were acquired by participating centres. All raw data (FCS files) were returned to the organising centre for centralised gating.					
	Detailed gating strategy	Samples were firstly gated on a live cell population (except Centre C). A lymphocyte gate based on size and granularity followed, before defining CD3+CD4-CD8+multimer+ cells. As the donors were known to be HIV seronegative, HIV-multimer^{+} populations were used as a negative control and test antigen multimer^{+} gates were set accordingly.					
	Representative data set<br>Post-IVS<br>Multimer<br>Phase II<br>Donor 5	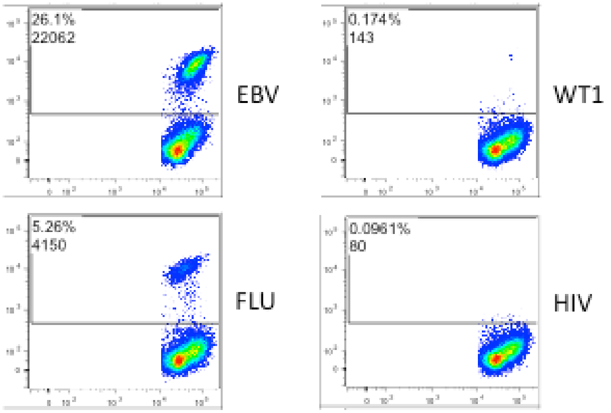					
	Review of raw data?	All dot plots (test and control) were examined by 3 independent analysts in a blinded fashion and scores were given based upon both the frequency and appearance of multimer^{+} T-cells - (2) a clustered population, (1) ambiguous population (0) clearly negative. A positive response was defined as a combined score of ≥5.					
	Mean % multimer^{+} T-cells<br>Donors 1-3 (I)<br>Donors 4-5 (II)	**Centre A**	**Centre B**	**Centre C**	**Centre D**	**Centre E**	**Centre F**
	**Phase I** –<br>FLU response	Ex vivo: 0.57<br>Post-IVS: 20.7	Ex vivo: 0.13<br>Post-IVS: 1.6	Ex vivo: 0.21<br>Post-IVS: 22.2	Ex vivo: 0.61<br>Post-IVS: 42.6	Ex vivo: 0.09<br>Post-IVS: 23.8	Ex vivo: N/A<br>Post-IVS: N/A
	**Phase I** –<br>CMV response	Ex vivo: ND<br>Post-IVS: ND	Ex vivo: ND<br>Post-IVS: ND	Ex vivo: ND<br>Post-IVS: 0.08	Ex vivo: ND<br>Post-IVS: 0.12	Ex vivo: ND<br>Post-IVS: ND	Ex vivo: N/A<br>Post-IVS: N/A

	**Phase I** – HIV response	Ex vivo: ND Post-IVS: ND	Ex vivo: ND Post-IVS: ND	Ex vivo: ND Post-IVS: ND	Ex vivo: ND Post-IVS: ND	Ex vivo: ND Post-IVS: ND	Ex vivo: N/A Post-IVS: N/A
	**Phase II** – EBV response	Ex vivo: N/A Post-IVS: 39.2	Ex vivo: N/A Post-IVS: 34.5	Ex vivo: N/A Post-IVS: 37.9	Ex vivo: N/A Post-IVS: 29.4	Ex vivo: N/A Post-IVS: 20.0	Ex vivo: N/A Post-IVS: 49.6
	**Phase II** – FLU response	Ex vivo: N/A Post-IVS: 2.5	Ex vivo: N/A Post-IVS: 2.7	Ex vivo: N/A Post-IVS: 1.7	Ex vivo: N/A Post-IVS: 2.1	Ex vivo: N/A Post-IVS: 0.5	Ex vivo: N/A Post-IVS: 2.3
	**Phase II** – WT1 response	Ex vivo: N/A Post-IVS: 0.12	Ex vivo: N/A Post-IVS: 0.10	Ex vivo: N/A Post-IVS: 0.09	Ex vivo: N/A Post-IVS: 0.09	Ex vivo: N/A Post-IVS: 0.06	Ex vivo: N/A Post-IVS: 0.06
	**Phase II** – HIV response	Ex vivo: N/A Post-IVS: ND	Ex vivo: N/A Post-IVS: ND	Ex vivo: N/A Post-IVS: ND	Ex vivo: N/A Post-IVS: ND	Ex vivo: N/A Post-IVS: ND	Ex vivo: N/A Post-IVS: ND
**Module 4 – Results (Multimer staining)**		**Centre A**	**Centre B**	**Centre C**	**Centre D**	**Centre E**	**Centre F**
**4A – Raw data**	Cut off specifications and # of tests OOS	Phase I, 1 replicate post-IVS, all donors	Phase II, 1 replicate post-IVS, donor 4	N/A	Phase I, ex vivo, donor 1	Phase I, 1 replicate, ex vivo, all donors	N/A
	Accessibility of raw data addressed?	Raw data may be made available upon request.					
**4B – Response determination**	Definition of positive reactivity (above background) incl. tests applied	Following review of data (see 3B), a score of ≥5 was defined as a positive response.					
	Response definition pre-defined or post-hoc	Post-hoc					
**Module 5**		**Centre A**	**Centre B**	**Centre C**	**Centre D**	**Centre E**	**Centre F**
**5A – General lab operation**	Guidance of lab operations	Exploratory research	Exploratory research	Exploratory research	Exploratory research and GCLP	Work to GCLP principles	GLP
**5B – Standardisation**	Status of protocols	SOP	Standardised protocols for MULTIMER staining and ELISPOT à all steps covered by SOPs	Established protocol	SOP	SOP	SOP
**5C – Qualification /validation**	Status of assays	ELISPOT: Optimised/qualified	ELISPOT and Multimer:	ELISPOT and Multimer:	ELISPOT: Validated Multimer: Optimised	ELISPOT and Multimer:	ELISPOT: Validated

		Multimer: Non-validated	Optimised and pre-validated	Optimised and non-validated		Optimised and validated	Multimer: Optimised

N/A, Not applicable; ND, Not Detected; NK, Not Known; P/S, Penicillin and Streptomycin; o/n, overnight; SOP, Standard Operating Procedure.

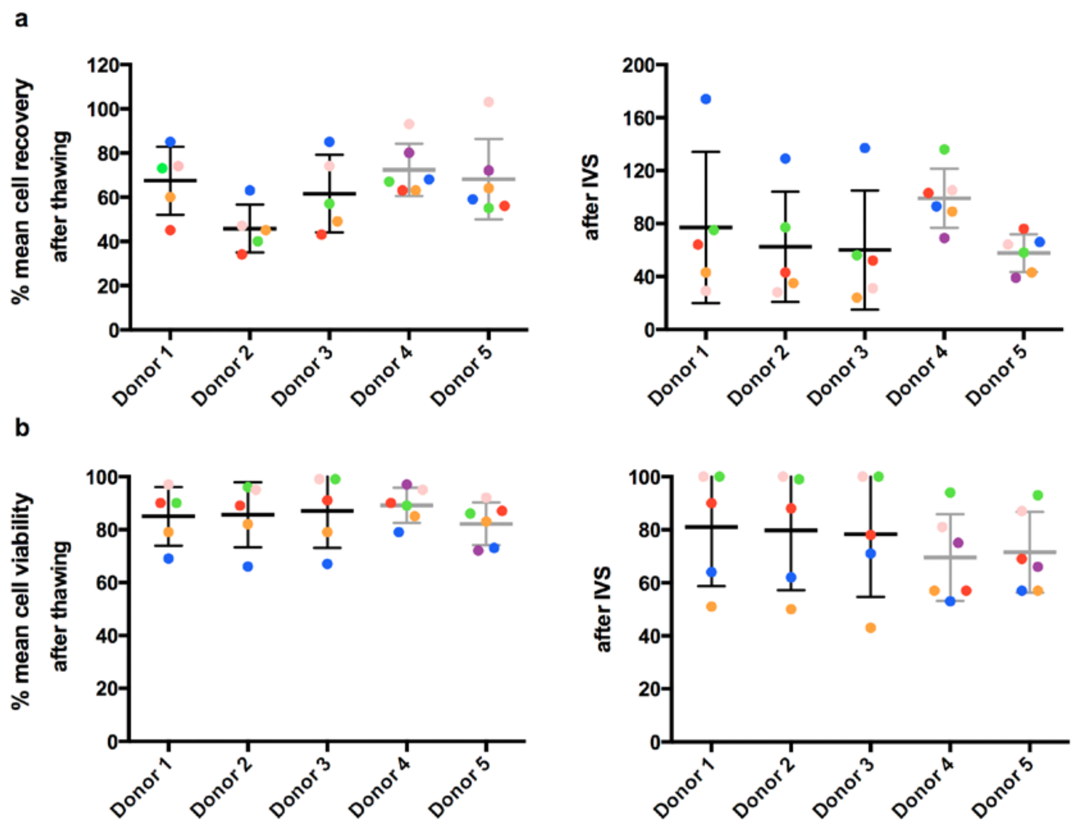

Supplementary Fig. 1 Cell recovery and cell viability upon thawing and post-IVS in Phase I and II Cell recovery (**a**) and cell viability (**b**) of 5 donors (1-5) were assessed after initial thawing (*left*) and post-IVS (*right*); data for each centre represent a mean of ≥3 repeats. Donors 1-3 and 4-5 featured in Phase I (*black error bars*) and II (*grey error bars*), respectively. Centre A, blue; Centre B, red; Centre C, green; Centre D, orange; Centre E, pink; Centre F, purple.

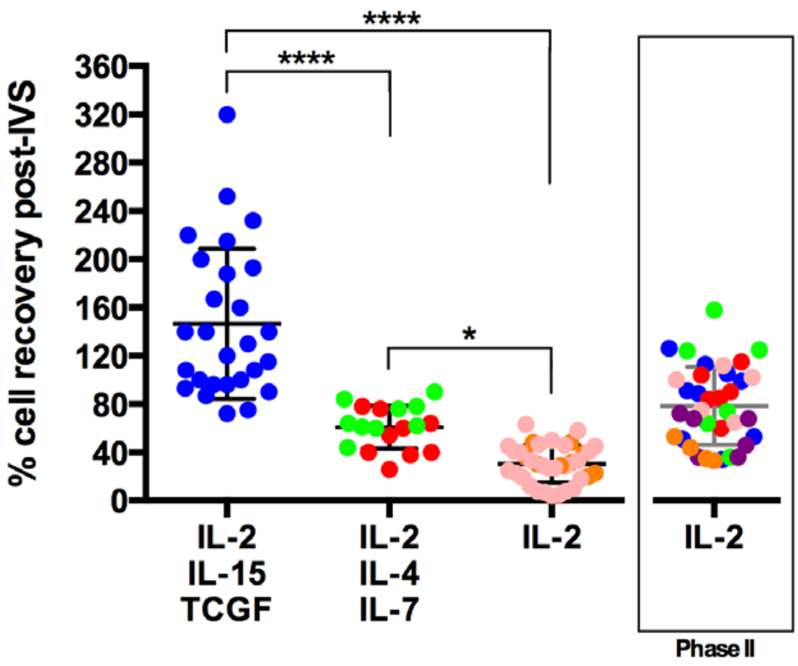

Supplementary Fig. 2 Effect of cytokines on cell recovery post-IVS in Phase I (and II) Cell recovery, relative to the cell number plated on day 1, of 3 donors (1-3) was assessed post-IVS in Phase I; data for each centre represent individual IVS experiments. For comparison, cell recovery post-harmonised IVS in Phase II is also shown for donors 4-5 (*boxed*). Centre A, blue; Centre B, red; Centre C, green; Centre D, orange; Centre E, pink; Centre F, purple. ****P<0.0001, *P<0.01

Supplementary Table 1
A list of HLA-A*0201-restricted epitopes for ELISPOT and multimer analyses

Antigen	Protein	Position	Sequence
Human CMV	pp65	495-503	NLVPMVATV
EBV	BMLF1	280-288	GLCTLVAML
FLU	M1	58-66	GILGFVFTL
HIV-1	Pol (RT)	476-484	ILKEPVHGV
-	WT1	37-45	VLDFAPPGA

Supplementary Table 2
Mean fold increase (± SD) of FLU-specific responses post-IVS as detected by ELISPOT and multimer staining for centres A-E in Phase I

		Centre A	Centre B	Centre C	Centre D	Centre E	Mean
ELISPOT	Donor 1	259.2 ± 25.8	29.2 ± 22.1	150.3 ± 20.3	190.8 ± 81.0	89.0 ± 93.2	135.5 ± 93.5
	Donor 2	116.4 ± 25.0	103.3 ± 151.8	122.0 ± 67.6	130.8 ± 29.0	83.4 ± 41.7	110.8 ± 70.7
	Donor 3	242.7 ±3.8	6.9 ± 1.4	105.1 ± 27.0	948.5 ± 249.9	160.8 ± 95.4	318.6 ± 382.1
	Mean	206.1 ± 18.2	46.5 ± 58.4	125.8 ± 38.3	423.3 ± 120.0	111.1 ± 76.7	185.1 ± 238.5
Multimer	Donor 1	74.4 ± 0.5	17.4 ± 10.7	225.5 ± 85.6	195.5 ± 215.8	251.8 ± 352.9	147.7 ± 163.0
	Donor 2	80.2 ± 3.5	18.2 ± 20.6	114.0 ± 91.0	101.0 ± 34.5	325.8 ± 441.6	116.3 ± 167.0
	Donor 3	8.9 ± 8.3	2.1 ± 0.1	22.3 ± 12.8	38.7 ± 21.8	70.2 ± 96.3	28.8 ± 38.9
	Mean	54.5 ± 4.1	12.6 ± 10.5	120.6 ± 63.1	111.7 ± 90.7	216.0 ± 297.0	98.1 ± 142.9

Supplementary Table 3

Inter-assay % coefficient of variation for FLU and CMV responses as detected by *ex vivo* and post-IVS ELISPOT and multimer staining for centres A-E in Phase I

			ELISPOT: Inter-assay %CV						Multimer: Inter-assay %CV					
			Centre A	Centre B	Centre C	Centre D	Centre E	Mean	Centre A	Centre B	Centre C	Centre D	Centre E	Mean
FLU	*Ex vivo*	Donor 1	24.0	23.2	14.4	38.0	23.3	24.6	25.1	24.2	14.4	87.0	1.1	30.4
		Donor 2	42.5	74.5	16.2	23.3	7.6	32.8	6.0	29.7	19.0	43.4	23.9	24.4
		Donor 3	112.0	122.6	55.4	22.7	9.9	64.5	85.2	30.9	56.1	47.9	4.7	45.0
		Mean	59.5	73.4	28.7	28.0	13.6	40.6	38.8	28.3	29.8	59.4	9.9	33.3
	Post-IVS	Donor 1	8.2	77.9	26.4	N/A[a]	74.1	46.7	1.5	46.8	48.7	32.2	87.8	43.4
		Donor 2	22.5	88.1	39.9	N/A[a]	44.7	48.8	1.0	90.4	65.0	6.6	83.5	49.3
		Donor 3	52.8	57.3	37.7	N/A[a]	54.7	50.6	107.0	42.4	32.0	40.0	86.9	61.7
		Mean	27.8	74.4	34.7	N/A[a]	57.8	48.7	36.5	59.9	48.6	26.3	86.1	51.5
CMV	*Ex vivo*	Donor 1	128.9	89.2	173.2	173.2	0.0	141.1	8.2	36.3	13.6	90.8	99.0	49.6
		Donor 2	21.6	173.2	86.6	86.6	0.0	92.0	22.7	48.7	40.1	42.2	31.5	37.0
		Donor 3	120.2	137.8	120.2	173.2	173.2	144.9	70.4	34.8	114.0	65.2	18.4	60.6
		Mean	90.2	133.4	126.7	144.3	57.7	126.0	33.8	39.9	55.9	66.1	49.6	49.1
	Post-IVS	Donor 1	141.4	29.6	124.8	173.2	0.0	117.3	2.6	72.2	65.8	164.0	47.2	70.4
		Donor 2	81.2	173.2	9.0	173.2	173.2	122.0	2.7	62.9	128.6	133.9	164.2	98.5
		Donor 3	141.4	0.0	173.2	173.2	89.8	144.4	2.0	46.8	34.1	154.2	60.5	59.5
		Mean	121.3	67.6	102.3	173.2	87.7	127.9	2.4	60.6	76.2	150.7	90.6	76.1

[a]Test wells were saturated with SFC too numerous to count; these wells were reported with a maximum spot number of 2000.
Not applicable, N/A

Supplementary Table 4
Inter-assay % coefficient of variation for EBV, FLU and WT1.37 responses as detected by post-harmonized IVS ELISPOT and multimer staining for centres A-F in Phase II

		ELISPOT: Inter-assay %CV							Multimer: Inter-assay %CV						
		Centre A	Centre B	Centre C	Centre D	Centre E	Centre F	Mean	Centre A	Centre B	Centre C	Centre D	Centre E	Centre F	Mean
EBV	Donor 4	8.3	2.8	32.4	72.9	18.2	47.2	30.3	37.3	16.0	15.6	42.2	24.0	22.2	26.2
	Donor 5	27.9	47.3	74.2	139.6	60.2	46.8	66.0	61.8	73.6	29.2	122.4	40.3	56.0	63.9
	Mean	18.1	25.1	53.3	106.3	39.2	47.0	48.2	49.6	44.8	22.4	82.3	32.2	39.1	45.1
FLU	Donor 4	30.6	22.8	107.7	36.0	23.1	48.5	44.8	42.6	29.7	49.5	44.6	75.4	23.5	44.2
	Donor 5	47.1	80.7	63.9	135.5	41.3	125.2	82.3	25.9	71.5	48.6	96.0	58.1	55.6	59.3
	Mean	38.9	51.8	85.8	85.8	32.2	86.9	63.6	34.3	50.6	49.1	70.3	66.8	39.6	51.8
WT1	Donor 4	N/A	N/A	N/A	N/A	N/A	N/A	N/A	142.3	23.1	66.3	19.6	16.6	96.1	60.7
	Donor 5	N/A	N/A	N/A	N/A	N/A	N/A	N/A	47.0	27.6	58.1	44.5	58.1	14.6	41.7
	Mean	N/A	N/A	N/A	N/A	N/A	N/A	N/A	94.7	25.4	62.2	32.1	37.4	55.4	51.2

Not applicable, N/A

Supplementary Table 5
Inter-lab % coefficient of variation for FLU and CMV responses as detected by *ex vivo* and post-IVS ELISPOT and multimer staining for centres A-E in Phase I

		ELISPOT: Inter-lab %CV				Multimer: Inter-lab %CV			
		**Donor 1**	**Donor 2**	**Donor 3**	**Mean**	**Donor 1**	**Donor 2**	**Donor 3**	**Mean**
**FLU**	***Ex vivo***	29.8	50.7	120.2	66.9	90.9	65.9	92.8	83.2
	**Post-IVS**	65.1	69.9	118.8	84.6	75.1	73.1	105.7	84.6
**CMV**	***Ex vivo***	170.7	141.6	177.3	163.2	133.3	102.8	108.9	115.0
	**Post-IVS**	221.9	240.9	188.9	217.2	324.0	149.0	246.5	239.8

Supplementary Table 6

Inter-lab % coefficient of variation for EBV, FLU and WT1 responses as detected by post-harmonized IVS ELISPOT and multimer staining for centres A-F in Phase II

	ELISPOT: Inter-lab %CV			Multimer: Inter-lab %CV		
	**Donor 4**	**Donor 5**	**Mean**	**Donor 4**	**Donor 5**	**Mean**
**EBV**	41.6	73.3	57.5	38.4	63.4	50.9
**FLU**	77.4	89.8	83.6	65.5	66.5	66.0
**WT1**	N/A	N/A	N/A	162.1	64.5	113.3

Not applicable, N/A

Supplementary Table 7
Correlation of *ex vivo* with post-IVS responses and ELISPOT with multimer responses from Phase I and II

		**ELISPOT** ***Ex vivo*** **: Post-IVS** (FLU)	**Multimer** ***Ex vivo*** **: Post-IVS** (FLU)	***Ex vivo*** **ELISPOT : Multimer** (FLU)	**Post-IVS** **ELISPOT : Multimer** (FLU/FLU&EBV)
**Phase I**	**Donor 1**	R^2 = 0.2459<br>P = 0.0714	R^2 = 0.0897<br>P = 0.3442	R^2 = 0.0860<br>P = 0.3310	R^2 = 0.5988<br>**P = 0.0012**
	**Donor 2**	R^2 = 0.5411<br>**P = 0.0027**	R^2 = 0.3882<br>P = 0.0229	R^2 = 0.1726<br>P = 0.1396	R^2 = 0.3829<br>P = 0.0183
	**Donor 3**	R^2 = 0.0203<br>P = 0.6274	R^2 = 0.2413<br>P = 0.0883	R^2 = 0.2794<br>P = 0.0520	R^2 = 0.5302<br>**P = 0.0031**
**Phase II**	**Donor 4**	N/A	N/A	N/A	R^2 = 0.7685<br>**P < 0.0001**
	**Donor 5**	N/A	N/A	N/A	R^2 = 0.3424<br>**P = 0.0002**
		R^2 = 0.3312<br>**P < 0.0001**	R^2 = 0.1244<br>P = 0.0299	R^2 = 0.1080<br>P = 0.0359	R^2 = 0.5113<br>**P < 0.0001**

Correlations were evaluated using the Pearson's correlation coefficient (R) and the coefficient of determination (R^2)
Significance testing was via the Student's T-test with a confidence level of 99% (< 0.01%)
Not applicable, N/A
